# Beta-Glycerophosphate-Induced ORAI1 Expression and Store Operated Ca^2+^ Entry in Megakaryocytes

**DOI:** 10.1038/s41598-020-58384-x

**Published:** 2020-02-03

**Authors:** Lisann Pelzl, Itishri Sahu, Ke Ma, David Heinzmann, Abdulla Al Mamun Bhuyan, Tamer al-Maghout, Basma Sukkar, Yamini Sharma, Irene Marini, Flaviana Rigoni, Ferruh Artunc, Hang Cao, Ravi Gutti, Jakob Voelkl, Burkert Pieske, Meinrad Gawaz, Tamam Bakchoul, Florian Lang

**Affiliations:** 1 Transfusion Medicine, Medical Faculty, Eberhard Karl University Tuebingen, Tuebingen, Germany; 2 Department of Internal Medicine III, Eberhard Karl University Tuebingen, Tuebingen, Germany; 3 Department of Biochemistry, School of Life Sciences, University of Hyderabad, Hyderabad, 500046 India; 4 Department of Internal Medicine IV, Eberhard Karl University Tuebingen, Tuebingen, Germany; 5 Institute for Physiology, Johannes Kepler University, Linz, Austria; 6 DZHK (German Centre for Cardiovascular Research), partner site Berlin, Berlin, Germany; 7 Department of Nephrology and Medical Intensive Care, Charité University Medicine, Berlin, Germany; 8 Berlin Institute of Health (BIH), Berlin, and Department of Internal Medicine and Cardiology, German Heart Center Berlin (DHZB), Berlin, Germany; 9 Centre for Clinical Transfusion Medicine, University Hospital of Tuebingen, Tuebingen, Germany; 10 Department of Vegetative and Clinical Physiology, Eberhard Karl University Tuebingen, Tuebingen, Germany; 11 Department of Internal Medicine and Cardiology, Campus Virchow Klinikum, Charité University Medicine, Berlin, Germany

**Keywords:** Glomerulus, End-stage renal disease

## Abstract

Impairment of renal phosphate elimination in chronic kidney disease (CKD) leads to enhanced plasma and tissue phosphate concentration, which in turn up-regulates transcription factor NFAT5 and serum & glucocorticoid-inducible kinase SGK1. The kinase upregulates ORAI1, a Ca^2+^-channel accomplishing store-operated Ca^2+^-entry (SOCE). ORAI1 is stimulated following intracellular store depletion by Ca^2+^-sensors STIM1 and/or STIM2. In megakaryocytes and blood platelets SOCE and thus ORAI1 are powerful regulators of activity. The present study explored whether the phosphate-donor ß-glycerophosphate augments NFAT5, ORAI1,2,3 and/or STIM1,2 expressions and thus SOCE in megakaryocytes. Human megakaryocytic Meg01cells were exposed to 2 mM of phosphate-donor ß-glycerophosphate for 24 hours. Platelets were isolated from blood samples of patients with impaired kidney function or control volunteers. Transcript levels were estimated utilizing q-RT-PCR, cytosolic Ca^2+^-concentration ([Ca^2+^]_i_) by Fura-2-fluorescence, and SOCE from increase of [Ca^2+^]_i_ following re-addition of extracellular Ca^2+^ after store depletion with thapsigargin (1 µM). NFAT5 and ORAI1 protein abundance was estimated with Western blots. As a result, ß-glycerophosphate increased NFAT5, ORAI1/2/3, STIM1/2 transcript levels, as well as SOCE. Transcript levels of NFAT5, SGK1, ORAI1/2/3, and STIM1/2 as well as NFAT5 and ORAI1 protein abundance were significantly higher in platelets isolated from patients with impaired kidney function than in platelets from control volunteers. In conclusion, phosphate-donor ß-glycerophosphate triggers a signaling cascade of NFAT5/SGK1/ORAI/STIM, thus up-regulating store-operated Ca^2+^-entry.

## Introduction

Compromised renal elimination of phosphate leads in chronic kidney disease (CKD) to increase of phosphate concentration in plasma and tissues which in turn triggers vascular calcification leading to cardiovascular events and the respective increases of morbidity and mortality^1–4^. Calcium deposition in the vascular wall involves osteo-/chrondrogenic reprogramming of vascular smooth muscle cells (VSMCs)^5–8^. The signaling includes up-regulation of the transcription factor NFAT5 (nuclear factor of activated T cells 5)^9–12^. NFAT5 was originally cloned as tonicity responsive enhancer binding protein (TonEBP) stimulated by hyperosmotic cell shrinkage^13,14^ and subsequently been shown to be enhanced in several disorders, such as diabetes^15^, inflammation^16^ and CKD^12^.

NFAT5-regulated genes include SGK1 (serum and glucocorticoid inducible kinase 1)^17^. SGK1 up-regulates the transcription factor NFκB (nuclear factor κB)^18^. NFκB triggers transcription of the Ca^2+^-channel ORAI1. Following depletion of intracellular Ca^2+^-stores ORAI1 is activated by Ca^2+^ sensing STIM (stromal interaction molecules)^18^. Opening of ORAI1 by STIM leads to the so-called store operated Ca^2+^-entry (SOCE)^18^, SOCE is a powerful mechanism activating blood platelets^19,20^. Genetic or pharmacological knockout of SGK1 down-regulates ORAI1^21^, blunts platelet activation^22,23^ and thus counteracts thrombosis^23^ and arteriosclerosis^24^. SGK1 and thus SGK1-dependent up-regulation of ORAI1 expression is stimulated by ischemia, oxidative stress, hyperglycemia, advanced glycation end products (AGEs) and several mediators including glucocorticoids, mineralocorticoids, transforming growth factor beta (TGFβ), interleukin 6 (IL-6), platelet-derived growth factor (PDGF), thrombin and endothelin^25^. By stimulating ORAI1 expression, upregulated SGK1 may increase the risk of thrombo-occlusive events in diabetes mellitus, inflammation, and chronic kidney disease^20,24,25^.

Platelets are released into the blood stream by megakaryocytes, which differentiate from hematopoietic progenitor cells in the bone marrow^26,27^. Megakaryocytes reorganize their cytoplasm into long proplatelet extensions that release platelets into the circulation^27^. Consequently, the proteins expressed in megakaryocytes are expected to be transferred into circulating blood platelets^27^. Recent observations revealed a powerful SGK1 dependent stimulation of ORAI1 expression by NFAT5 overexpression in megakaryocytes^28^.

In view of those observations we hypothesized that the phosphate-donor ß-glycerophosphate mimicking enhanced extracellular phosphate concentration may up-regulate the expression of NFAT5 in megakaryocytes, and that NFAT5 enhances the expression of SGK1, ORAI1 and STIM1 and/or STIM2. Considering that the abundance of the respective proteins in circulating platelets is a function of protein synthesis in megakaryocytes, enhanced extracellular phosphate may up-regulate the expression of NFAT5, SGK1, ORAI1 and STIM1 and/or STIM2 in circulating blood platelets. To the best of our knowledge, however, nothing is hitherto known on the contribution of NFAT5 to the regulation of megakaryocyte or platelet function in CKD patients.

The present study thus explored whether NFAT5, SGK1, ORAI1, ORAI2, ORAI3 STIM1 and/or STIM2 expression in megakaryocytes is sensitive to phosphate-donor ß-glycerophosphate and altered in patients with CKD incl. dialysis-dependency.

## Results

**Figure 1:**
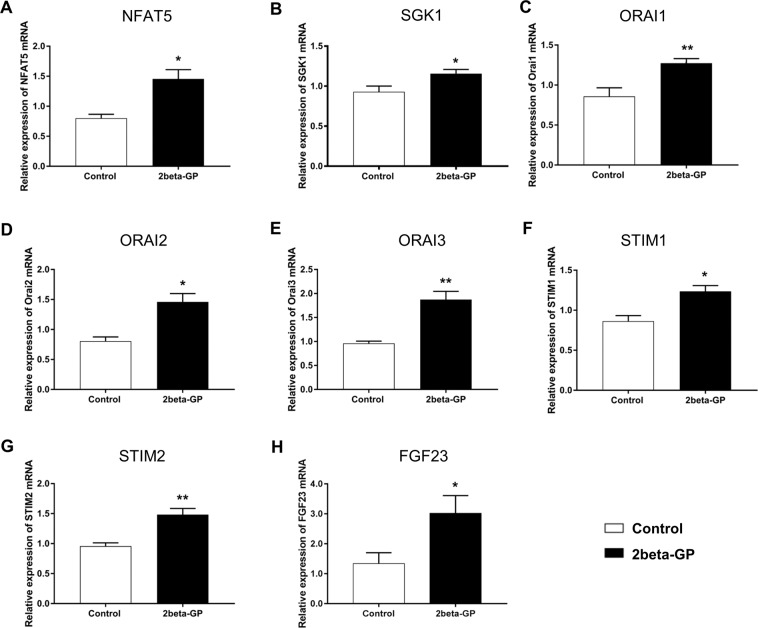
ß-glycerophosphate-sensitive NFAT5, SGK1, ORAI1, ORAI2, STIM1, STIM2 and FGF23 transcription in megakaryocytes (**A–E**). Arithmetic means (±SEM, n = 4–7) of NFAT5 (**A**), SGK1 (**B**), ORAI1 (**C**), ORAI2 (**D**), ORAI3 (**E**), STIM1 (**F**), STIM2 (**G**) and FGF23 (**H**) transcript levels in megakaryocytes without (white bars) and with (black bars) prior exposure to 2 mM ß-glycerophosphate for 24 hours. *(p < 0.05), **(p < 0.01) indicates statistically significant difference to respective value without prior ß-glycerophosphate treatment (Student’s t-test).

As the transcription factor NFAT5 is known to upregulate expression of SGK1^17^ which in turn activates transcription factor NFkB with upregulation of ORAI and STIM isoform expression^18^, the present study explored whether the phosphate donor ß-glycerophosphate modifies the transcript levels of the transcription factor nuclear factor of activated T cells 5 (NFAT5), of the NFAT5-regulated serum & glucocorticoid inducible kinase 1 (SGK1), of the SGK1-sensitive Ca^2+^ release activated ion channels ORAI1, ORAI2 and ORAI3 as well as of the ORAI activating Ca^2+^ sensor isoforms STIM1 and STIM2. As illustrated in Fig. 1, 24 hours exposure of human megakaryocytes to 2 mM ß-glycerophosphate was followed by a significant increase in the transcript levels of NFAT5, SGK1, ORAI1, ORAI2, ORAI3, STIM1, and STIM2 (Fig. 1A–G). As illustrated in Fig. 1H, exposure to 2 mM ß-glycerophosphate further increases the transcription of fibroblast growth factor 23 (FGF23), a gene sensitive to ORAI1-dependent Ca^2+^ entry into UMR106 bone cells^29^.

**Figure 2:**
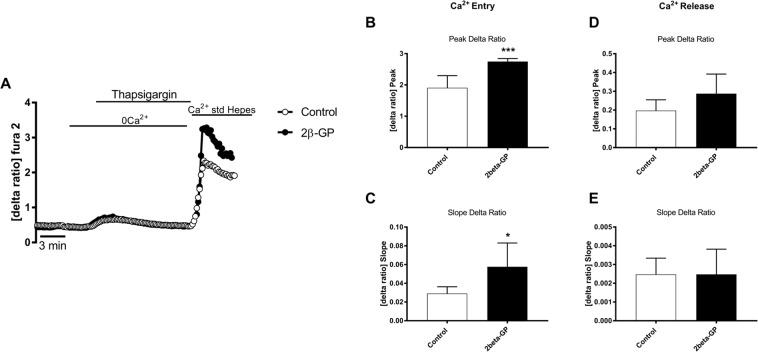
ß-glycerophosphate-sensitive intracellular Ca^2+^ release and store-operated Ca^2+^ entry (SOCE) in Meg01 cells. (**A**). Representative tracings of Fura-2 fluorescence-ratio in fluorescence spectrometry before and following extracellular Ca^2+^ removal and addition of thapsigargin (1 µM), as well as re-addition of extracellular Ca^2+^ in Meg01 cells without (control, white circles) or with (black circles) prior exposure to 2 mM ß-glycerophosphate for 24 hours. (**B,C**). Arithmetic means (±SEM, n = 56–52 cells from 7 groups) of peak (**B**) and slope (**C**) increase of fura-2-fluorescence-ratio following addition of thapsigargin (1 µM) in Meg01 cells without (control, white bars) or with (black bars) prior exposure to 2 mM ß-glycerophosphate for 24 hours. (**D,E**). Arithmetic means (±SEM, n = 56–52 cells from 7 groups) of peak (**D**) and slope (**E**) increase of fura-2-fluorescence-ratio following re-addition of extracellular Ca^2+^ in Meg01cells without (control, white bars) or with (black bars) prior exposure to 2 mM ß-glycerophosphate for 24 hours. *(p < 0.05), ***(p < 0.0005) indicates statistically significant difference to respective value without prior ß-glycerophosphate treatment (Student’s t- test).

In order to test, whether the enhanced expression of ORAI and STIM isoforms is followed by the respective alterations of Ca^2+^ signaling, Fura2 fluorescence was utilized to determine the cytosolic Ca^2+^ activity ([Ca^2+^]_i_). The 340 nm/380 nm ratio reflecting [Ca^2+^]_i_ was, prior to store depletion, similar in ß-glycerophosphate treated (0.425 ± 0.030, n = 7) and in untreated (0.459 ± 0.046, n = 7) megakaryocytes. For determination of SOCE, cells were exposed to thapsigargin (1 µM), a sarco-/endoplasmic reticulum Ca^2+^/ATPase (SERCA) inhibitor, and Ca^2+^-free solutions to deplete intracellular Ca^2+^ stores. In the following extracellular Ca^2+^ was re-added in the continued presence of thapsigargin to quantify SOCE from the increase of [Ca^2+^]_I_ (Fig. 2A). As illustrated in Fig. 2A,C–E, ß-glycerophosphate pretreatment significantly increased both peak and slope of SOCE. SOCE was significantly decreased by the selective ORAI1 inhibitor MRS1845 (10 µM) (Supplementary Fig. 3). The 340 nm/380 nm ratio was similar prior to (0.444 ± 0.016, n = 4) and 3 min following (0.495 ± 0.022, n = 4) acute administration of 2 mM ß-glycerophosphate in the presence of extracellular Ca^2+^ (Supplementary Fig. 4). Moreover, a 30 min pretreatment with 2 mM ß-glycerophosphate did not significantly modify SOCE (Supplementary Fig. 5).

**Figure 3:**
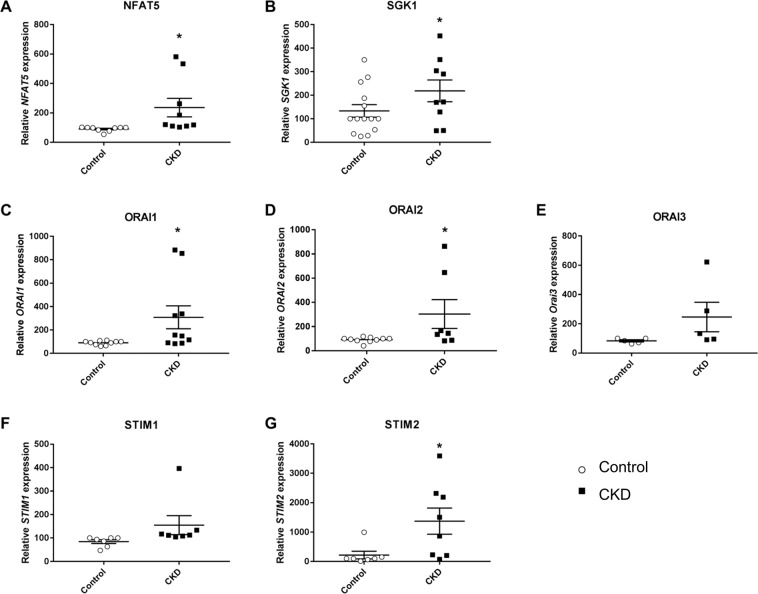
NFAT5, SGK1, ORAI1, STIM1, ORAI2 transcript levels in platelets from control volunteers and patients with chronic kidney disease. (**A–G**). Single values and arithmetic means (±SEM) of (**A**) NFAT5, (**B**) SGK1, **(C)** ORAI1, (**D**) ORAI2, (**E**) ORAI3, (**F**) STIM1, and (**G**) STIM2 transcript levels in platelets drawn from control volunteers (white circles) or patients with impaired kidney function (black squares).

**Figure 4:**
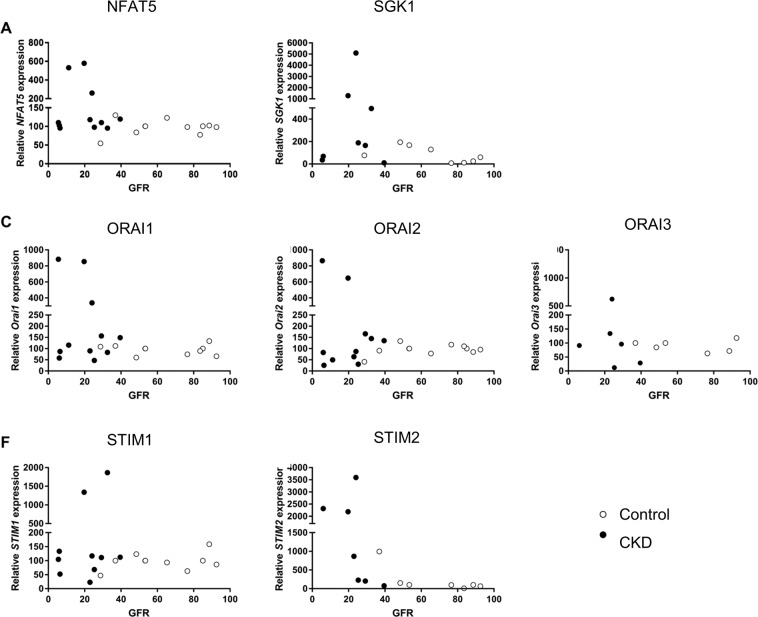
Correlation between GFR and NFAT5, SGK1, ORAI1, STIM1, ORAI2 transcript levels in platelets from control volunteers and patients with chronic kidney disease. (**A–G**). Single values of (**A**) NFAT5, (**B**) SGK1, **(C)** ORAI1, (**D**) ORAI2 (**E**), Orai3 (**F**), STIM1, and (**G**) STIM2 transcript levels in platelets drawn from control volunteers (white circles) or patients with impaired kidney function (black circles) as a function of GFR.

In order to test, whether the observed upregulation of NFAT5, SGK1, ORAI1, ORAI2, STIM1 and STIM2 by ß-glycerophosphate in megakaryocytes leads to the respective alterations of transcript levels in circulating blood platelets, the cells were isolated from patients with impaired kidney function and control volunteers with normal kidney function. Supplementary Fig. 2 displays creatinine plasma levels, glomerular filtration rate (GFR), as well as numbers of leukocytes and platelets in blood from control volunteers and patients with impaired kidney function. As illustrated in Fig. 3, the transcript levels of NFAT5, SGK1, ORAI1, ORAI2, and STIM2 were significantly higher in blood platelets from patients with impaired kidney function than in platelets from control volunteers. As illustrated in Fig. 4, NFAT5, SGK1, ORAI isoforms and STIM isoforms transcript levels were negatively correlated with GFR, i.e. a decline of GFR was associated with enhanced transcript levels of NFAT5, SGK1, ORAI1, ORAI2, STIM1 and STIM2.

**Figure 5:**
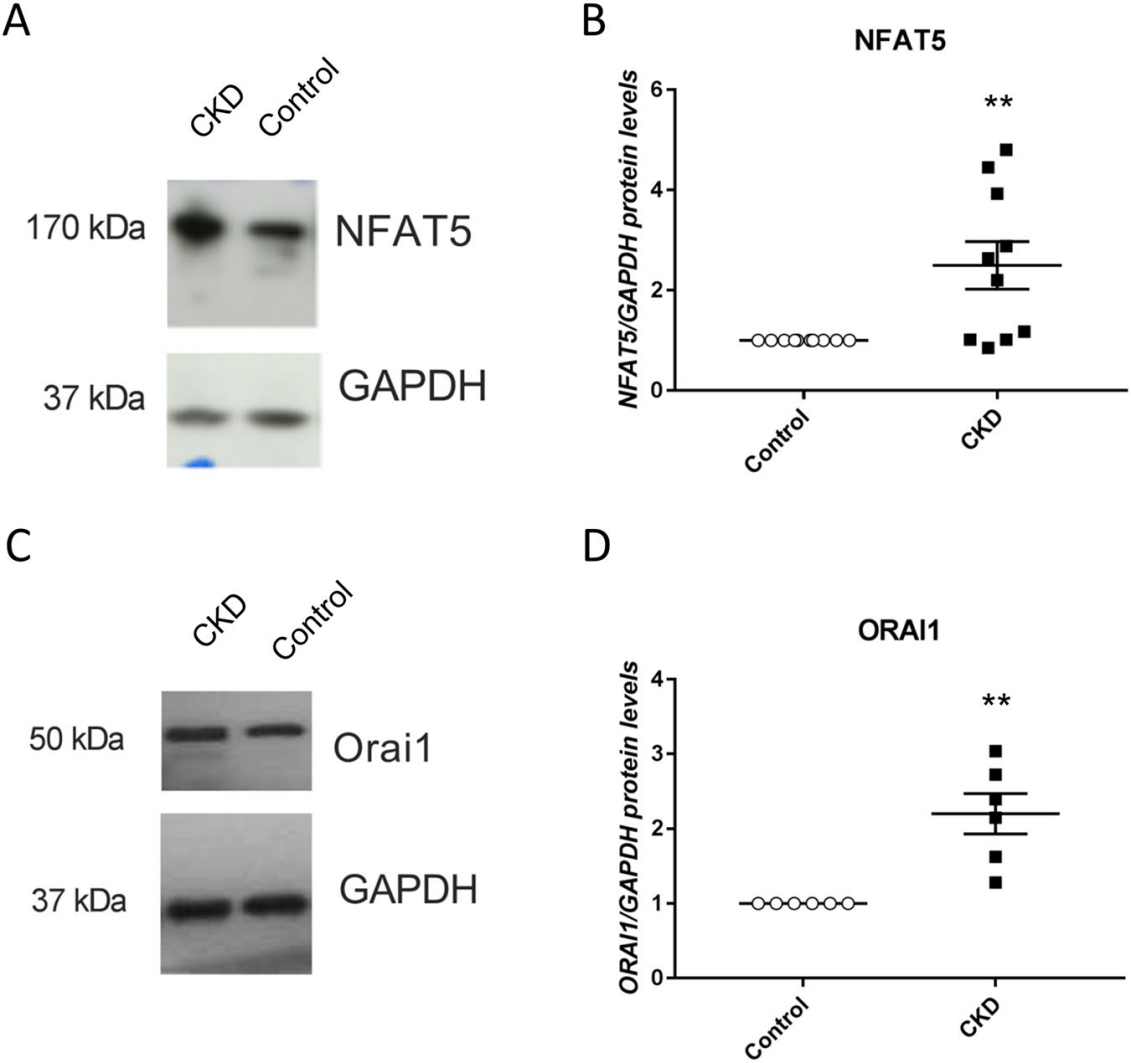
NFAT5 and ORAI1 protein abundance in platelets from control volunteers and patients with chronic kidney disease. (**A–C**). Original Western blots of (**A**) NFAT5 and **(C)** ORAI1 protein abundance in platelets drawn from control volunteers (**B–D**). Arithmetic means (±SEM) of (**B**) NFAT5 and **(D)** ORAI1 protein abundance in platelets drawn from control volunteers (white circles) or patients with impaired kidney function (black squares).

Western blotting was employed in order to test, whether the enhanced NFAT5 and ORAI1 transcript levels in patients with impaired kidney function are paralleled by the respective alterations of protein abundance. As illustrated in Fig. 5, both, NFAT5 and ORAI1 protein abundance was significantly higher in platelets from patients with impaired kidney function than in platelets from control volunteers.

## Discussion

The present study discloses a novel effect of the phosphate donor ß-glycerophosphate in megakaryocytes, i.e. the upregulation of ORAI1, ORAI2, ORAI3, STIM1 and STIM2 expression. ORAI1 is a Ca^2+^ channel^22^ shown to accomplish store operated Ca^2+^ entry (SOCE) in multiple cell types^30^ including platelets^20^ and megakaryocytes^22^. To the best of our knowledge, an effect of phosphate or of phosphate donor ß-glycerophosphate on ORAI, STIM and their isoforms has never been shown before in megakaryocytes or platelets.

The effect of phosphate-donor ß-glycerophosphate is disrupted by pharmacological inhibition of SGK1 and is thus presumably due to upregulation of SGK1 by NFAT5. NFAT5 has previously been shown to increase the expression of SGK1^17^, which is known to trigger the degradation of the inhibitor protein IκBα thus allowing nuclear translocation of the transcription factor NFκB^18^. Genes up-regulated by NFκB include ORAI1^18^.

In view of the upregulation of NFAT5, SGK1, ORAI1, STIM1 and STIM2 expression in megakaryocytes by the phosphate donor ß-glycerophosphate, the transcript levels of NFAT5, SGK1, ORAI1, ORAI2, ORAI3, STIM1 and STIM2 were expected to be enhanced in platelets from patients with impaired kidney function. As a matter of fact, the transcript levels of each, NFAT5, SGK1, ORAI1, ORAI2, STIM1and STIM2 were significantly higher in platelets isolated from patients with impaired kidney function than in platelets isolated from control volunteers. As shown for NFAT5 and ORAI1, the increase of transcript levels is paralleled by the respective increase of protein abundance. NFAT5 expression has been shown to be upregulated by patients with advanced CKD incl. dialysis-dependency in other cell types^12^, but not in megakaryocytes or blood platelets.

The upregulation of ORAI1 and STIM2 in platelets of CKD patients is expected to sensitize the platelets for activators^31^. The presently observed stimulation of ORAI1 and STIM2 could thus contribute to the known high risk of cardiac infarction and stroke in CKD patients^32,33^. Excessive activation of blood platelets is well known to enhance the risk of cardiac infarction and stroke^34^. Additional effort is needed to define the contribution of phosphate-sensitive ORAI1 and STIM2 expression in blood platelets to the enhanced cardiovascular risk in patients with advanced CKD.

As shown for other cell types, NFAT5 is up-regulated in further clinical disorders, such as dehydration^11^, diabetes mellitus^15^ and inflammatory disease^16^. In those conditions the enhanced NFAT5 expression may lead to stimulation of SGK1 expression with subsequent upregulation of ORAI1, STIM1 and STIM2 in megakaryocytes and sensitization of blood platelets to activating stimuli. The signalling may again involve SGK1-sensitive degradation of the inhibitor protein IκBα thus allowing nuclear translocation of the transcription factor NFκB.

ORAI1 expression and thus SOCE may be sensitive to phosphate or ß-glycerophosphate in other cell types. The ORAI^35–39^ and STIM^40–44^ isoforms accomplish store operated Ca^2+^ entry (SOCE) in a myriad of cell types. Alterations of cytosolic Ca^2+^ activity in turn contribute to the regulation of diverse cellular functions including excitation, exocytosis, migration, cell proliferation and cell death^45–49^. Notably, ORAI1 and STIM1 are expressed in lymphocytes thus participating in the orchestration of immune response and inflammation^50,51^. The overexpression of ORAI1 and STIM1 in tumor cells contributes to cancer growth^52–56^. Future experimental effort will be required to define the pathophysiological impact of phosphate sensitive ORAI and STIM expression on inflammation and malignancy in CKD.

Genes previously shown to be upregulated by NFAT5, SGK1, ORAI1, ORAI2, STIM1 and STIM2 in UMR106 cells include FGF23^29^. Here we demonstrate that the phosphate donor ß-glycerophosphate upregulates FGF23 in megakaryocytes. In view of the putative influence of SOCE on FGF23 transcription in UMR106 cells^29^, it is tempting to speculate that the observed signaling contributes to the phosphate-induced FGF23 release from bone. Whether ORAI1 expression and function is sensitive to phosphate in UMR106 cells remains, however, to be shown.

**Figure 6:**
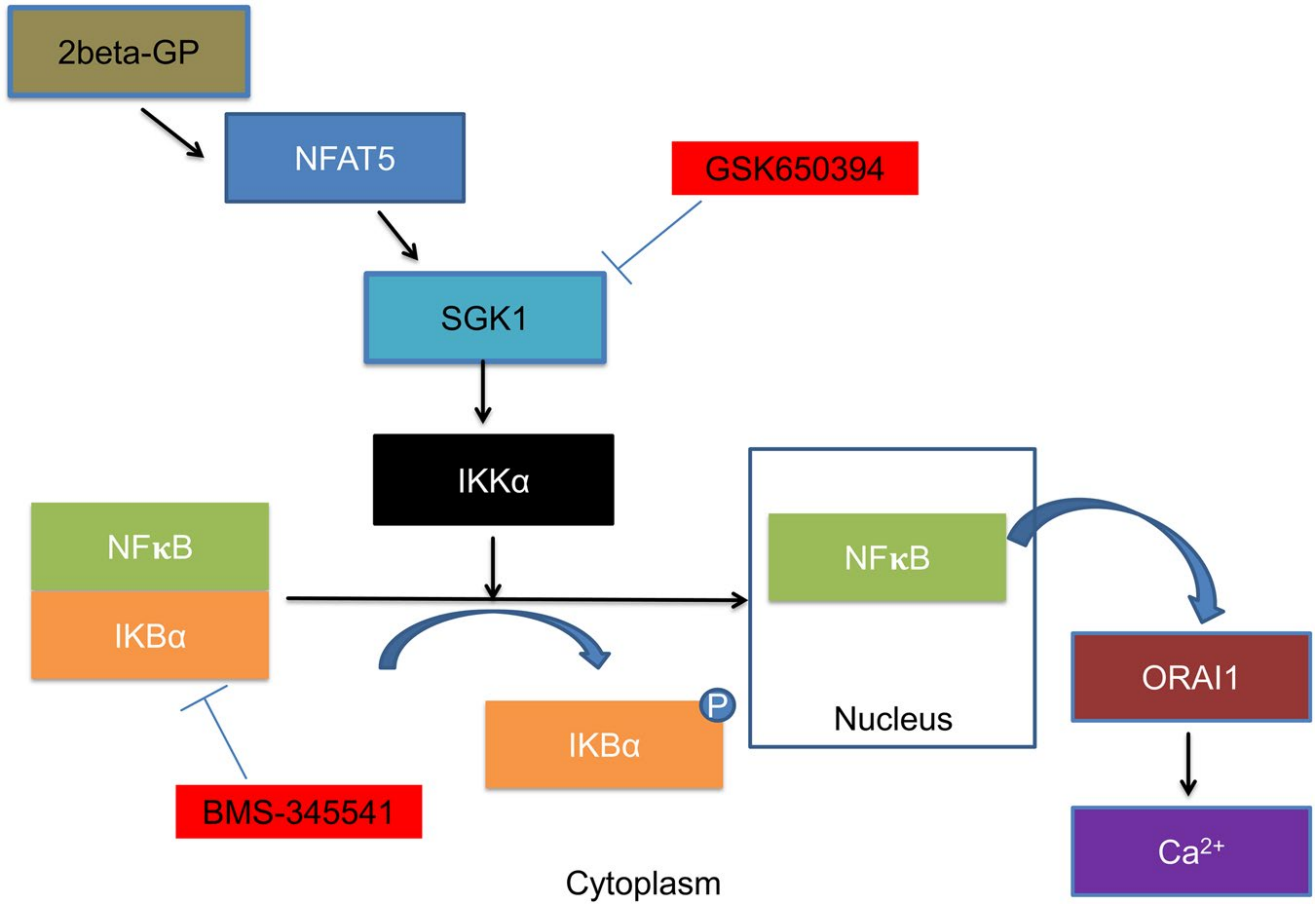
Diagram depicting the signaling events generated by treatment of Meg01 cells with ß-glycerophosphate (2beta-GP).

In conclusion, the phosphate donor ß-glycerophosphate stimulates the expression of NFAT5, SGK1, ORAI and STIM isoforms and thus store operated Ca^2+^ entry (SOCE) into megakaryocytes (Fig. 6). Accordingly, NFAT5, SGK1, ORAI and STIM isoform expression is enhanced in platelets of patients with impaired kidney function and could thus contribute to the enhanced cardiovascular risk in those patients.

## Materials and Methods

### Patients and volunteers

11 patients (8♂, 3♀) with impaired kidney function and 12 volunteers with normal kidney function (9♂, 3♀) have been enrolled in the study. The patients were recruited from the Department of Internal Medicine, University Hospital Tuebingen. The study was performed in accordance to the approval by ethics committee of the University of Tübingen (270/2011BO1) and in accordance with the Declaration of Helsinki. Informed written consent was provided by both, volunteers and patients. Clinical details of the patients are compiled in Table 1.

**Table 1:** Recruited patients with advanced CKD incl. dialysis-dependency and control volunteers.

Parameters	All Patients	Renal impairment	Control	p value
	n = 23 (100%)	n = 11 (48%)	n = 12 (52%)	
**Clinical characteristics**				
	Age	68.5 ± 3.4	72.4 ± 2.8	0.38
	Creatinine	4.2 ± 0.85	1.1 ± 0.08	0.0011
	GFR	19.2 ± 3.1	65.1 ± 5.7	0.0001
	CRP	1.3 ± 0.4	2.0 ± 0.7	0.4098
	Hb	11.6 ± 0.5	11.9 ± 0.6	0.7057
	WBC	8517 ± 726	7779 ± 409	0.3756
	platelets	197.9 ± 17.8	214.8 ± 18.1	0.5156
	female	3 (27%)	3 (25%)	1
	Dialysis	4 (36%)	0 (0%)	0.0373
	Diabetes	6 (55%)	5 (42%)	0.6843
	Hypertension	10 (91%)	10 (83%)	1
**Medication**				
	Betablockers	8 (73%)	10 (83%)	0.6404
	Statin	9 (82%)	10 (83%)	1
	ACE-Inh.	4 (36%)	4 (33%)	1
	AT-1 Inh.	2 (18%)	3 (25%)	1
	ASA	6 (55%)	7 (58%)	1
	Clopidogrel	3 (27%)	2 (17%)	0.6404
	Ticagrelor	2 (18%)	5 (42%)	0.3707
	Phenprocoumon	0 (0%)	1 (8%)	1
	DOAK	1 (9%)	1 (8%)	1

### Preparation of human platelets

Venous blood was drawn into tubes containing acid/citrate/dextrose buffer, from the antecubital vein of volunteers and patients with impaired kidney function. Platelet-rich plasma (PRP) was removed by centrifugation at 430 × g for 20 min. PRP was centrifuged at 900 × g for 10 min after adding Tyrodes/HEPES buffer (2.5 mmol/liter HEPES, 1 mmol/liter KCl, 2.5 mmol/liter NaHCO_3_, 150 mmol/liter NaCl, 0.36 mmol/liter NaH_2_PO_4_, 5.5 mmol/liter glucose, and 1 mg/ml BSA, pH 6.5). The platelets were resuspended after removing supernatant, in 200 µl of Tyrodes/HEPES buffer (supplemented with 1 mmol/liter CaCl_2,_ pH 7.4) and used for RNA/protein isolation. ~98% purity of the platelet preparation was obtained as determined by a Haematology Analyser (Sysmex KX219, Sysmex Germany GmbH).

### Cell culture

Human megakaryocytic cells (Meg01) from ATCC (American Type Culture Collection, Manassas, VA, USA) were cultured in RPMI 1640 (Roswell Park Memorial Institute medium, Gibco Thermo Fischer Scientific, Paisley, United Kingdom) containing 10% FBS and 1% Penicillin/Streptomycin in humidified incubator at 37 °C and 5% CO_2_. Where indicated, the cells were exposed to 2 mM ß-glycerophosphate (Sigma, Steinheim, Germany) for 24 hours in the absence and presence of ORAI1 inhibitor MRS1845 (10 µM) (Tocris, Bristol, United Kingdom). In the analysis of vascular calcification, phosphate donor ß-glycerophosphate is widely used as substitute for phosphate and well established stimulator of tissue calcification^57–59^.

### Quantitative PCR

To determine transcript levels of NFAT5, ORAI1, ORAI2, ORAI3, STIM1, STIM2 and FGF23 total RNA was extracted according to manufacturer’s instructions with TriFast (Peqlab, Erlangen, Germany)^60–64^. DNAse digestion was performed to avoid DNA contamination and was followed by reverse transcription using random hexamers (Promega, Manheim, Germany) and GoScript™ reverse transcription system (Promega, Manheim, Germany). Real-time polymerase chain reaction (RT-PCR) amplification of the respective genes were set up in a total volume of 20 µl using 40 ng of cDNA, 500 nM forward and reverse primer and 2x GoTaq® qPCR Master Mix (Promega, Hilden, Germany) following manufacturer’s protocol. Cycling conditions were as follows: initial denaturation at 95 °C for 2 minutes, followed by 40 cycles of 95 °C for 15 seconds, 55 °C for 15 seconds and 68 °C for 20 seconds. For amplification the following primers were used (5′- > 3′ orientation) (Invitrogen, Carlsbad, CA, USA)):

NFAT5

fw: GAGCAGAGCTGCAGTAT

rev: AGCTGAGAAAGCACATAG

GAPDH:

fw: TGAGTACGTCGTGGAGTCCAC

rev: GTGCTAAGCAGTTGGTGGTG

ORAI1:

fw: CGTATCTAGAATGCATCCGGAGCC

rev: CAGCCACTATGCCTAGGTCGACTAGC

ORAI2:

fw: CAGCTCCGGGAAGGAACGTC

rev: CTCCATCCCATCTCCTTGCG

ORAI3:

fw:CTTCCAATCTCCCACGGTCC

rev:GTTCCTGCTTGTAGCGGTCT

SGK1:

fw:TTCCTATCGCAGTGTTTCAGTTCTT

rev:CACACTCACACGACGGTTCAC

STIM1:

fw: CCTCGGTACCATCCATGTTGTAGCA

rev: GCGAAAGCTTACGCTAAAATGGTGTCT

STIM2:

fw: CAAGTTGCCCTGCGCTTTAT

rev: ATTCACTTTTGCACGCACCG

FGF23:

fw: ATGAGCGTCCTCAGAGCCTA

rev: AGACGTCGTACCCGTTTTCC

Melting curves were analysed to confirm PCR product specificity. CFX96 Real-Time System (BioRad, Munich, Germany) was used to perform real-time PCR amplifications and all experiments were done in duplicate. The house-keeping gene Glyceraldehyde 3-phosphate dehydrogenase (GAPDH) was amplified to standardize the amount of sample RNA.

### Western blotting

Protein abundance of NFAT5, ORAI1, and GAPDH was determined by Western blotting^60–64^. After isolation of platelets from control donors and patients with impaired kidney function, platelets were centrifuged for 5 minutes at 600 g and 4 °C. The pellet was washed with ice cold PBS and suspended in 50 μl ice-cold RIPA lysis buffer (Cell Signalling Technology, USA) containing Protease Inhibitor Cocktail (Sigma-Aldrich, Taufkirchen, Germany). After centrifugation (20,000 g, 4 °C for 20 minutes) the supernatant was taken to determine protein concentration using the Bradford assay (BioRad, München, Germany). For Western blotting 50 µg of protein were electro-transferred onto a nitrocellulose membrane after electrophoresis using 12% SDS- PAGE and blocked with 5% milk in TBST at room temperature for 1 h. The membranes were incubated with primary anti-NFAT5 antibody (1:1000, Novus Biologicals), anti-ORAI1 antibody (1:1000, Proteintech, Chicago, USA) and anti-GAPDH antibody (1:1000, Cell Signaling, Danvers, USA) at 4 °C overnight. After washing (TBST), the blots were incubated with secondary anti-rabbit antibody conjugated with horseradish peroxidase (1:1000, Cell Signaling, Danvers, USA) for 1 h at room temperature. Protein bands were detected after additional washes (TBST) with an ECL detection reagent (Amersham, Freiburg, Germany). For densitometry image analysis, western blots were scanned and analyzed by ImageJ software (NIH, USA), and the results are shown as the ratio of total protein to GAPDH normalized to the control group. Protein-Marker VI (Peqlab, Erlangen, Germany) was used as reference to assign the right protein size.

### Calcium measurements in megakaryocytes

To determine cytosolic Ca^2+^ concentration ([Ca^2+^]_i_), Fura-2/AM fluorescence was utilized^60–65^. Cells were preincubated for 15–30 minutes with Fura-2/AM (2 µM, Invitrogen, Goettingen, Germany) at 37 °C and excited alternatively at 340 nm and 380 nm in an inverted phase-contrast microscope (Axiovert 100, Zeiss, Oberkochen, Germany) through an objective (Fluor 40×/1.30 oil). At 505 nm, the emitted fluorescence intensity was recorded. Data (6/minute) were acquired using a computer software Metafluor (Universal Imaging, Downingtown, USA). To estimate cytosolic Ca^2+^ activity, a ratiometer (340 nm/380 nm) based analysis was employed. SOCE was determined following extracellular Ca^2+^ removal causing store depletion and subsequent Ca^2+^ re-addition in constant presence of SERCA inhibitor thapsigargin (1 µM, Invitrogen,Goettingen, Germany). For quantification of Ca^2+^ entry, the slope (delta ratio/s) and peak (delta ratio) were determined following re-addition of Ca^2+^. Experiments were performed with Ringer’s solution containing (in mM): 125 NaCl, 5 KCl, 1 CaCl_2_, 32 HEPES, 2 Na_2_HPO_4_, 1.2 MgSO_4_, 5 glucose, pH 7.4. Ca^2+^-free conditions were achieved by using Ca^2+^-free Ringer solution containing (in mM): 125 NaCl, 5 KCl, 1.2 MgSO_4_, 2 Na_2_HPO_4_, 32 HEPES, 0.5 EGTA, 5 glucose, pH 7.4.

### Statistical analysis

Data are provided as means ± SEM, *n* represents the number of independent experiments (i.e. in fluorescence experiments the number of dishes measured). Patient data are shown in scatter plots to illustrate the scatter of the data between patients. All data were tested for significance using unpaired t-test (Student’s t-test) or ANOVA. Results with p < 0.05 were considered statistically significant.

### Ethical permission

The study was approved by the ethics committee of the University of Tuebingen (270/2011BO1) and has been executed in accordance with the Declaration of Helsinki. Both, volunteers and patients provided informed written consent. The data have been presented at a conference^66^.

## Supplementary information

supplementary information.

## References

[1] Blacher J, Guerin AP, Pannier B, Marchais SJ, London GM (2001). Arterial calcifications, arterial stiffness, and cardiovascular risk in end-stage renal disease. Hypertension.

[2] London GM (2003). Arterial media calcification in end-stage renal disease: impact on all-cause and cardiovascular mortality. Nephrol. Dial. Transpl..

[3] Foley RN, Parfrey PS, Sarnak MJ (1998). Epidemiology of cardiovascular disease in chronic renal disease. J. Am. Soc. Nephrol..

[4] Mizobuchi M, Towler D, Slatopolsky E (2009). Vascular calcification: the killer of patients with chronic kidney disease. J. Am. Soc. Nephrol..

[5] Kapustin AN (2015). Vascular smooth muscle cell calcification is mediated by regulated exosome secretion. Circ. Res..

[6] Lang F, Ritz E, Alesutan I, Voelkl J (2014). Impact of aldosterone on osteoinductive signaling and vascular calcification. Nephron Physiol..

[7] Lang F, Ritz E, Voelkl J, Alesutan I (2013). Vascular calcification–is aldosterone a culprit?. Nephrol. Dial. Transpl..

[8] Steitz SA (2001). Smooth muscle cell phenotypic transition associated with calcification: upregulation of Cbfa1 and downregulation of smooth muscle lineage markers. Circ. Res..

[9] Alesutan I (2015). Inhibition of Phosphate-Induced Vascular Smooth Muscle Cell Osteo-/Chondrogenic Signaling and Calcification by Bafilomycin A1 and Methylamine. Kidney Blood Press. Res..

[10] Feger M (2015). Inhibitory effect of NH4Cl treatment on renal Tgfss1 signaling following unilateral ureteral obstruction. Cell Physiol. Biochem..

[11] Lang F, Guelinckx I, Lemetais G, Melander O (2017). Two Liters a Day Keep the Doctor Away? Considerations on the Pathophysiology of Suboptimal Fluid Intake in the Common Population. Kidney Blood Press. Res..

[12] Leibrock CB (2015). NH_4_Cl Treatment Prevents Tissue Calcification in Klotho Deficiency. J. Am. Soc. Nephrol..

[13] Handler JS, Kwon HM (2001). Transcriptional regulation by changes in tonicity. Kidney Int..

[14] Zhou X (2016). How do kinases contribute to tonicity-dependent regulation of the transcription factor NFAT5?. World J. Nephrol..

[15] Hernandez-Ochoa EO (2012). Elevated extracellular glucose and uncontrolled type 1 diabetes enhance NFAT5 signaling and disrupt the transverse tubular network in mouse skeletal muscle. Exp. Biol. Med..

[16] Neuhofer W (2010). Role of NFAT5 in inflammatory disorders associated with osmotic stress. Curr. Genomics.

[17] Chen S (2009). Tonicity-dependent induction of Sgk1 expression has a potential role in dehydration-induced natriuresis in rodents. J. Clin. Invest..

[18] Lang F, Shumilina E (2013). Regulation of ion channels by the serum- and glucocorticoid-inducible kinase SGK1. FASEB J..

[19] Braun A (2009). Orai1 (CRACM1) is the platelet SOC channel and essential for pathological thrombus formation. Blood.

[20] Lang F, Munzer P, Gawaz M, Borst O (2013). Regulation of STIM1/Orai1-dependent Ca2+ signalling in platelets. Thrombosis Haemost..

[21] Eylenstein A (2011). Stimulation of Ca2+-channel Orai1/STIM1 by serum- and glucocorticoid-inducible kinase 1 (SGK1). FASEB J..

[22] Borst O (2012). The serum- and glucocorticoid-inducible kinase 1 (SGK1) influences platelet calcium signaling and function by regulation of Orai1 expression in megakaryocytes. Blood.

[23] Lang F, Gawaz M, Borst O (2015). The serum- & glucocorticoid-inducible kinase in the regulation of platelet function. Acta physiologica.

[24] Borst O (2015). Pivotal role of serum- and glucocorticoid-inducible kinase 1 in vascular inflammation and atherogenesis. Arterioscler. Thromb. Vasc. Biol..

[25] Lang F, Voelkl J (2013). Therapeutic potential of serum and glucocorticoid inducible kinase inhibition. Expert. Opin. investigational drugs.

[26] Balduini A (2011). *In vitro* megakaryocyte differentiation and proplatelet formation in Ph-negative classical myeloproliferative neoplasms: distinct patterns in the different clinical phenotypes. PLoS One.

[27] Golfier S (2010). Shaping of terminal megakaryocyte differentiation and proplatelet development by sphingosine-1-phosphate receptor S1P4. FASEB J..

[28] Sahu I (2017). NFAT5-sensitive Orai1 expression and store-operated Ca(2+) entry in megakaryocytes. FASEB J..

[29] Glosse P (2018). AMP-activated kinase is a regulator of fibroblast growth factor 23 production. Kidney Int..

[30] Parekh AB (2010). Store-operated CRAC channels: function in health and disease. Nat.Rev.Drug Discov..

[31] Berna-Erro A, Jardin I, Smani T, Rosado JA (2016). Regulation of Platelet Function by Orai, STIM and TRP. Adv. Exp. Med. Biol..

[32] Moody WE, Edwards NC, Chue CD, Ferro CJ, Townend JN (2013). Arterial disease in chronic kidney disease. Heart.

[33] Webster AC, Nagler EV, Morton RL, Masson P (2017). Chronic Kidney Disease. Lancet.

[34] Renga B, Scavizzi F (2017). Platelets and cardiovascular risk. Acta Cardiol..

[35] Prakriya M (2006). Orai1 is an essential pore subunit of the CRAC channel. Nat..

[36] Putney JW (2007). New molecular players in capacitative Ca2+ entry. J. Cell Sci..

[37] Vig M (2006). CRACM1 is a plasma membrane protein essential for store-operated Ca2+ entry. Sci..

[38] Yeromin AV (2006). Molecular identification of the CRAC channel by altered ion selectivity in a mutant of Orai. Nat..

[39] Zhang SL (2008). Store-dependent and -independent modes regulating Ca2+ release-activated Ca2+ channel activity of human Orai1 and Orai3. J. Biol. Chem..

[40] Fahrner M (2009). Mechanistic view on domains mediating STIM1-Orai coupling. Immunol. Rev..

[41] Peinelt C (2006). Amplification of CRAC current by STIM1 and CRACM1 (Orai1). Nat. Cell Biol..

[42] Penna A (2008). The CRAC channel consists of a tetramer formed by Stim-induced dimerization of Orai dimers. Nat..

[43] Smyth JT (2010). Activation and regulation of store-operated calcium entry. J. Cell Mol. Med..

[44] Zhang SL (2005). STIM1 is a Ca2+ sensor that activates CRAC channels and migrates from the Ca2+ store to the plasma membrane. Nat..

[45] Becchetti A, Arcangeli A (2010). Integrins and ion channels in cell migration: implications for neuronal development, wound healing and metastatic spread. Adv. Exp. Med. Biol..

[46] Burgoyne RD (2007). Neuronal calcium sensor proteins: generating diversity in neuronal Ca2+ signalling. Nat. Rev. Neurosci..

[47] Orrenius S, Zhivotovsky B, Nicotera P (2003). Regulation of cell death: the calcium-apoptosis link. Nat. Rev. Mol. Cell Biol..

[48] Roderick HL, Cook SJ (2008). Ca2+ signalling checkpoints in cancer: remodelling Ca2+ for cancer cell proliferation and survival. Nat. Rev. Cancer.

[49] Salter RD, Watkins SC (2009). Dendritic cell altered states: what role for calcium?. Immunol. Rev..

[50] Hwang DY (2014). Genetic polymorphisms of ORAI1 and chronic kidney disease in Taiwanese population. Biomed. Res. Int..

[51] Shaw PJ, Feske S (2012). Regulation of lymphocyte function by ORAI and STIM proteins in infection and autoimmunity. J. Physiol..

[52] Bergmeier W, Weidinger C, Zee I, Feske S (2013). Emerging roles of store-operated Ca(2)(+) entry through STIM and ORAI proteins in immunity, hemostasis and cancer. Channels.

[53] Capiod T (2013). The need for calcium channels in cell proliferation. Recent. Pat. Anticancer. Drug. Discov..

[54] Courjaret R, Machaca K (2012). STIM and Orai in cellular proliferation and division. Front. Biosci..

[55] Moccia F (2012). Store-dependent Ca(2+) entry in endothelial progenitor cells as a perspective tool to enhance cell-based therapy and adverse tumour vascularization. Curr. Med. Chem..

[56] Prevarskaya N, Skryma R, Shuba Y (2011). Calcium in tumour metastasis: new roles for known actors. Nat. Rev. Cancer.

[57] Giachelli CM (2001). Vascular calcification and inorganic phosphate. Am. J. Kidney Dis..

[58] Moe SM, Chen NX (2004). Pathophysiology of vascular calcification in chronic kidney disease. Circ. Res..

[59] Shioi A (1995). Beta-glycerophosphate accelerates calcification in cultured bovine vascular smooth muscle cells. Arterioscler. Thromb. Vasc. Biol..

[60] Abdelazeem KNM (2019). Upregulation of Orai1 and STIM1 expression as well as store-operated Ca(2+) entry in ovary carcinoma cells by placental growth factor. Biochem. Biophys. Res. Commun..

[61] Ma K (2019). Phosphate-induced ORAI1 expression and store-operated Ca(2+) entry in aortic smooth muscle cells. J. Mol. Med..

[62] Zhang S (2017). Epigallocatechin-3-gallate (EGCG) up-regulates miR-15b expression thus attenuating store operated calcium entry (SOCE) into murine CD4+ T cells and human leukaemic T cell lymphoblasts. Oncotarget.

[63] Pelzl L (2017). Lithium Sensitive ORAI1 Expression, Store Operated Ca(2+) Entry and Suicidal Death of Neurons in Chorea-Acanthocytosis. Sci. Rep..

[64] Sukkar B (2018). Inhibition of Lithium Sensitive Orai1/ STIM1 Expression and Store Operated Ca2+ Entry in Chorea-Acanthocytosis Neurons by NF-kappaB Inhibitor Wogonin. Cell Physiol. Biochem..

[65] Schmid E (2012). SGK3 regulates Ca(2+) entry and migration of dendritic cells. Cell Physiol. Biochem..

[66] Pelzl, L. *et al*. Phosphate-induced ORAI1 Expression and Store Operated Ca2+ Entry in Megakaryocytes. *Hamostaseologie* **39**, SY05-02-AB, 10.1055/s-0039-1680097 (2019).

