# Supplementary material for: Beta-Glycerophosphate-Induced ORAI1 Expression and Store Operated Ca2+ Entry in Megakaryocytes
Source: Sci Rep. 2020 Feb 3;10:1728. doi: 10.1038/s41598-020-58384-x (PMC6997179; doi:10.1038/s41598-020-58384-x)
Supplement: Supplementary file 1 — supplementary information. [file 41598_2020_58384_MOESM1_ESM.docx]

**Beta-Glycerophosphate-Induced ORAI1 Expression and Store Operated Ca^2+^ Entry in Megakaryocytes**

Lisann Pelzl^1^*, Itishri Sahu^2,3^*, Ke Ma^2^, David Heinzmann^2^, Abdulla Al Mamun Bhuyan^2^, Tamer al-Maghout^2^, Basma Sukkar^2^, Yamini Sharma^2^, Irene Marini^1^, Flaviana Rigoni^1^, Ferruh Artunc^4^, Hang Cao^2^, Ravi Gutti^3^, Jakob Voelkl^5,6,7^, Burkert Pieske^6,7,8^, Meinrad Gawaz^2^, Tamam Bakchoul^1,9^, Florian Lang^10^

^1^Transfusion Medicine, Medical Faculty, Eberhard Karl University Tübingen, Germany,

^2^Department of Internal Medicine III, Eberhard Karl University Tübingen, Germany,

^3^Department of Biochemistry, School of Life Sciences, University of Hyderabad, Hyderabad 500046, India,

^4^Department of Internal Medicine IV, Eberhard Karl University Tübingen, Germany,

^5^Institute for Physiology, Johannes Kepler University, Linz, Austria

^6^DZHK (German Centre for Cardiovascular Research), partner site Berlin, Berlin, Germany

^7^Department of Nephrology and Medical Intensive Care, Charité University Medicine, Berlin, Germany,

^8^Berlin Institute of Health (BIH), Berlin, and Department of Internal Medicine and Cardiology, German Heart Center Berlin (DHZB), Berlin, Germany,

^9^Centre for Clinical Transfusion Medicine, University Hospital of Tübingen, Germany,

^10^Department of Vegetative and Clinical Physiology, Eberhard Karl University Tübingen, Germany

*contributed equally and thus share first authorship


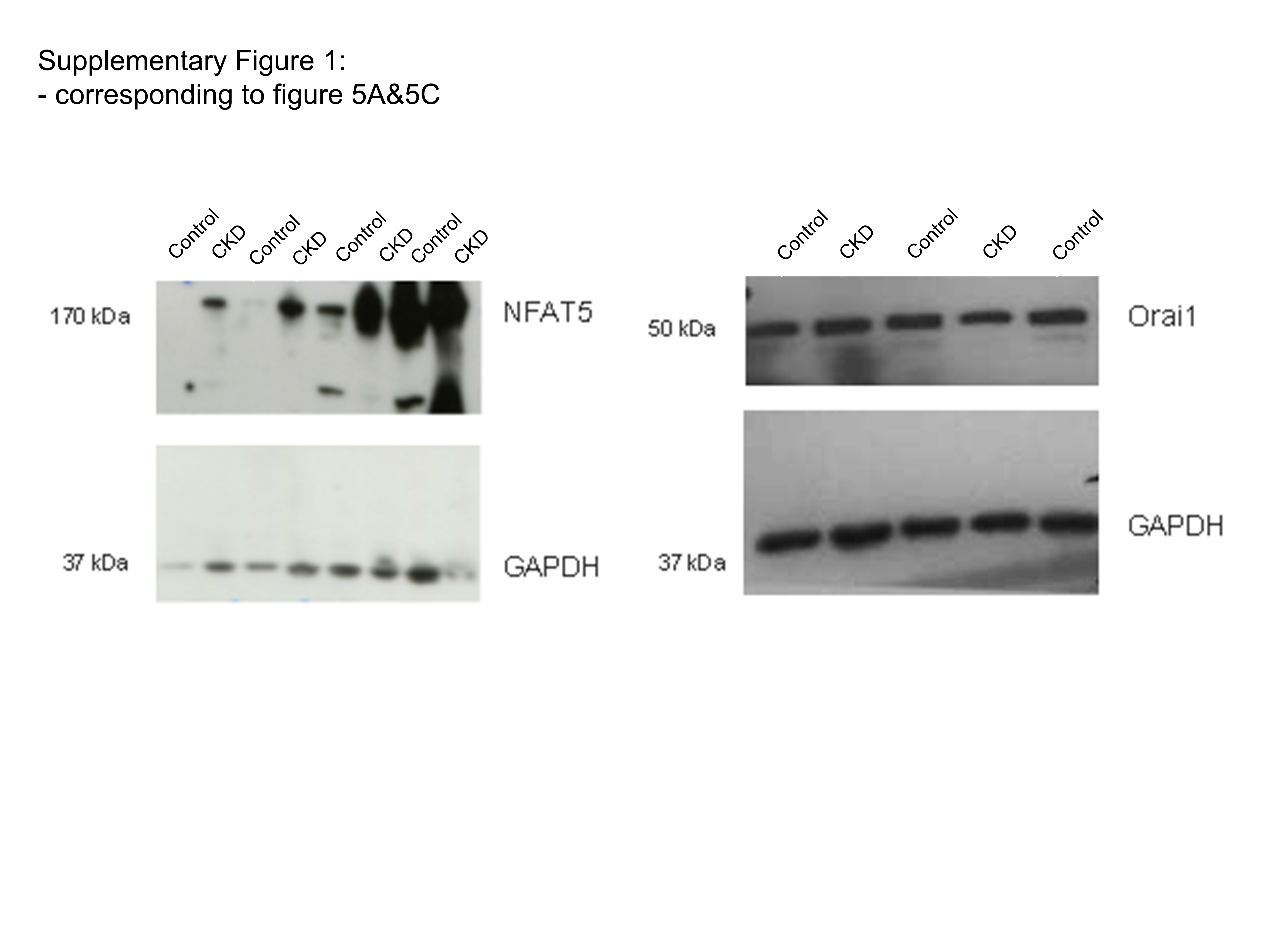


**Supplementary Figure 1 corresponding to figure 5**

NFAT5 and ORAI1 protein abundance in platelets from control volunteers and patients with impaired kidney function. A-B. Original Complete Western blots of (A) NFAT5 and (B) ORAI1 protein abundance in platelets drawn from control volunteers.


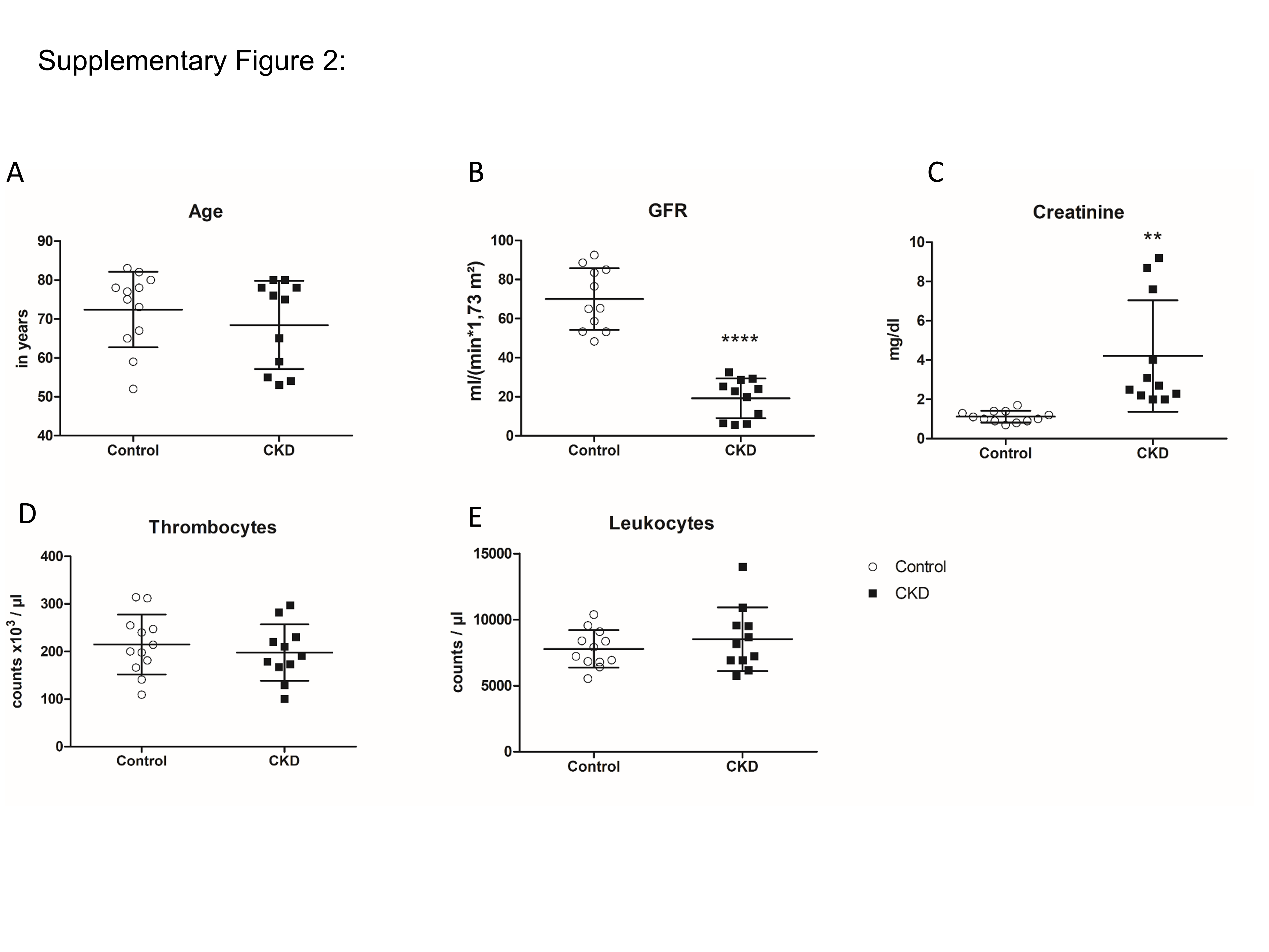


**Supplementary Figure 2. Age, creatinine plasma level, GFR, leukocyte number and platelet number in blood from control volunteers and patients** with impaired kidney function

**A-E.** Single values and arithmetic means (± SEM) of (**A**) age, (**B**) plasma creatinine, (**C**) GFR, (**D**) leukocyte number and (**E**) platelet number in blood drawn from control volunteers (white circles) or patients with impaired kidney function (black circles).

**(p<0.01), ****(p<0.0001) indicates statistically significant difference to respective value in control volunteers (Student’s t-test).


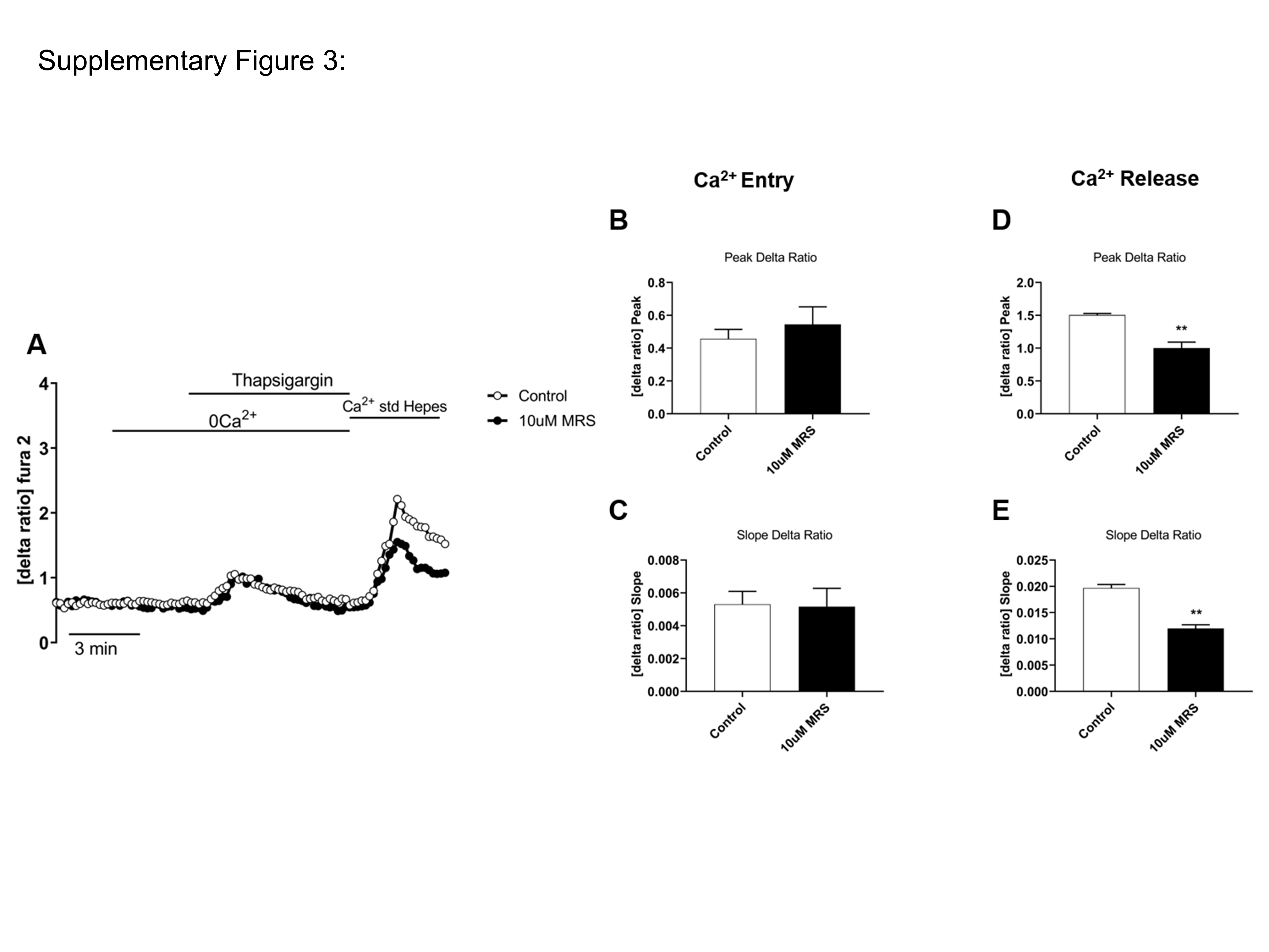


**Supplementary Figure 3**

**Effect of ORAI1 inhibitor MRS1845 on intracellular Ca^2+^ release and store-operated Ca^2+^ entry (SOCE) in Meg01 cells.**

**A.** Representative tracings of Fura-2 fluorescence ratio in fluorescence spectrometry before and following extracellular Ca^2+^ removal and addition of thapsigargin (1 µM), as well as re-addition of extracellular Ca^2+^ in Meg01 cells without (Control, white circles) or with (MRS, black circles) presence of ORAI1 inhibitor MRS1845.

**B,C.** Arithmetic means ± SEM (n = 25-35 cells from 4 groups) of slope (**B**) and peak (**C**) increase of fura-2 fluorescence ratio following addition of thapsigargin (1 µM) in Meg01 cells without (Control, white bars) or with (MRS, black bars) presence of ORAI1 inhibitor MRS1845 (10 µM).

**D,E.** Arithmetic means ± SEM (n = 25-35 cells from 4 groups) of slope (**D**) and peak (**E**) increase of fura-2 fluorescence ratio following re-addition of extracellular Ca^2+^ in Meg01 cells without (Control, white bars) or with (MRS, black bars) presence of ORAI1 inhibitor MRS1845 (10 µM).

**(p<0.05) indicates statistically significant difference to control group.


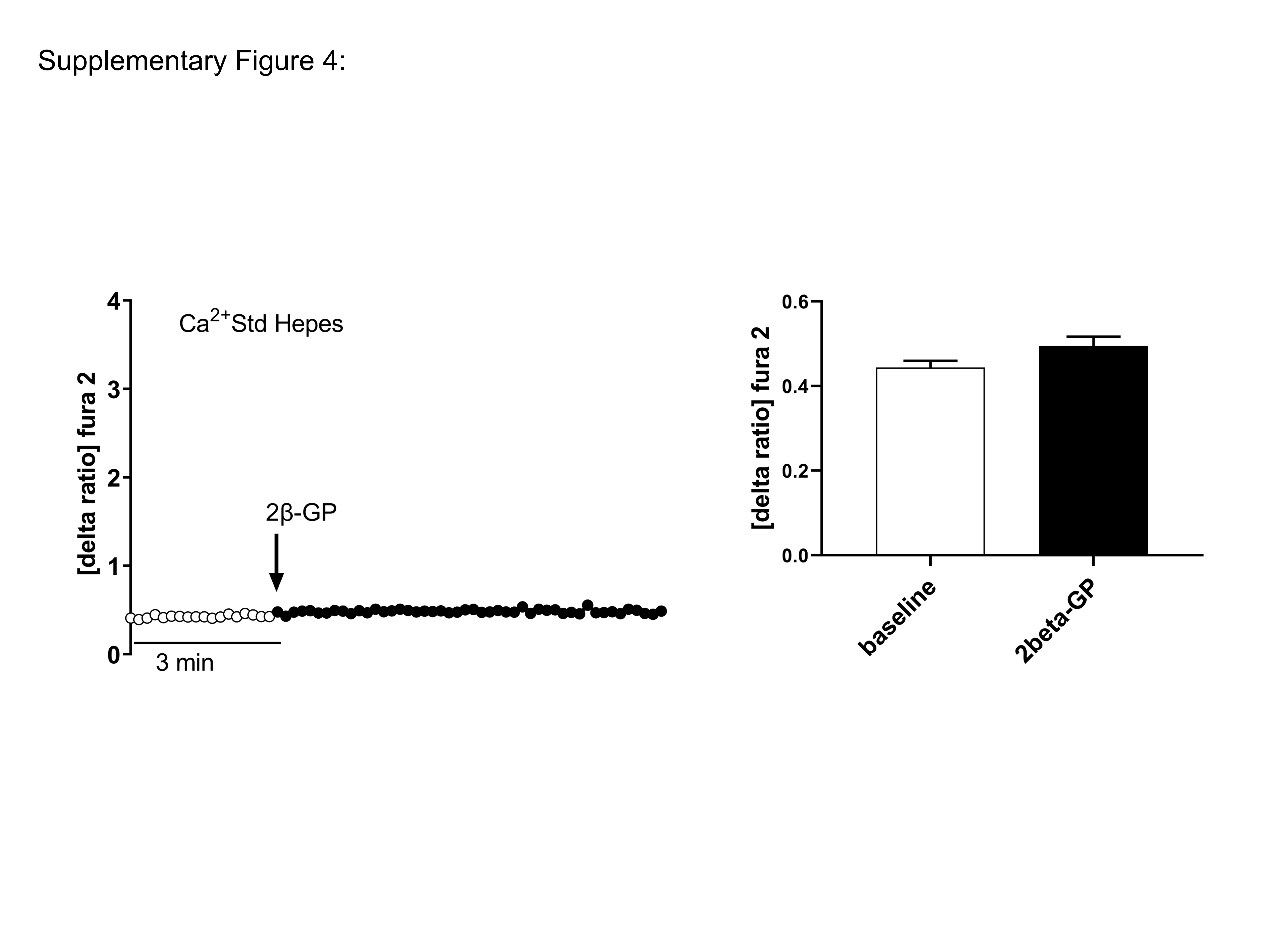


**Supplementary Figure 4**

**Acute effect of ß-glycerophosphate on intracellular Ca^2+^ in Meg01 cells.**

**A.** Representative tracings of Fura-2 fluorescence ratio in fluorescence spectrometry before and following 2mM ß-glycerophosphate treatment

**B.** Arithmetic means ± SEM (n = 25-35 cells from 4 groups) of prior to (baseline) and 3 min following acute administration of 2 mM ß-glycerophosphate (2beta-GP) in the presence of extracellular Ca^2+^.


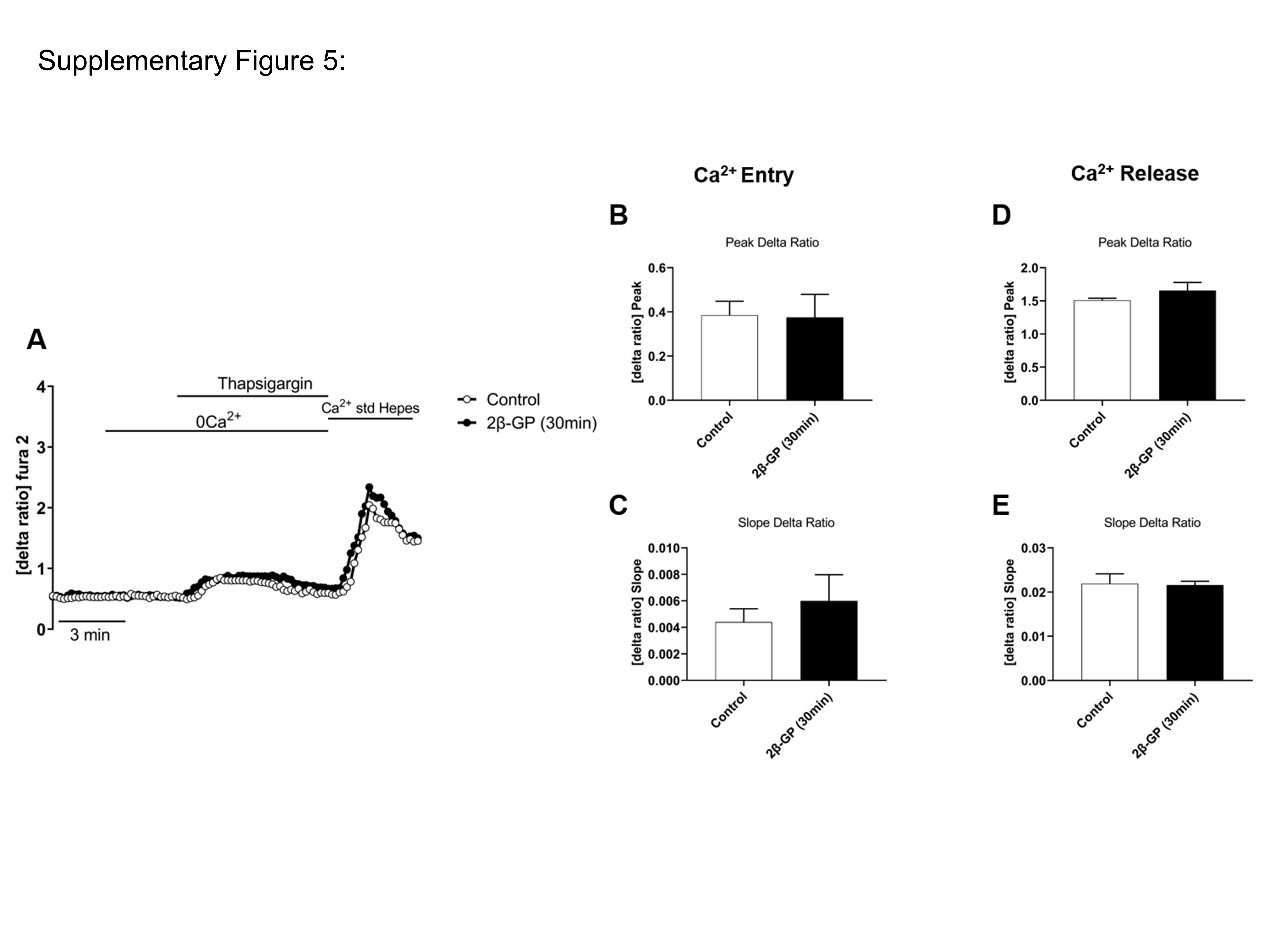


**Supplementary Figure 5**

**Effect of a 30 min exposure to ß-glycerophosphate on intracellular Ca^2+^ release and store-operated Ca^2+^ entry (SOCE) in Meg01 cells.**

**A.** Representative tracings of Fura-2 fluorescence ratio in fluorescence spectrometry before and following extracellular Ca^2+^ removal and addition of thapsigargin (1 µM), as well as re-addition of extracellular Ca^2+^ in Meg01 cells without (Control, white circles) or with β-glycerophosphate (black circles) for 30 min.

**B,C.** Arithmetic means ± SEM (n = 30-36 cells from 4 groups) of peak (**B**) and slope (**C**) increase of fura-2 fluorescence ratio following addition of thapsigargin (1 µM) in HAoSMCs without (Control, white bars) or with 2 mM ß-glycerophosphate (black bars) for 30 min.

**D,E.** Arithmetic means ± SEM (n = 30-36 cells from 4 groups) of peak (**D**) and slope (**E**) increase of fura-2 fluorescence ratio following re-addition of extracellular Ca^2+^ in HAoSMCs without (Control, white bars) or with (black bars) 2 mM ß-glycerophosphate pretreatment for 30 min.
